# Institutional barriers to clinical trial exploration experienced by the Latinx community

**DOI:** 10.1007/s13187-022-02259-4

**Published:** 2023-04-29

**Authors:** Sabrina Sandoval, Ringo K. Leung, France Nguyen-Grozavu, Regina M. Wang, Georgia Robins Sadler

**Affiliations:** 1 San Diego Moores Cancer Center, University of California San Diego, La Jolla, CA 92093 USA; 2 Herbert Wertheim School of Public Health and Human Longevity Science, University of California, San Diego, USA; 3 Department of Surgery, San Diego School of Medicine, University of California, San Diego, USA

**Keywords:** Access, Barriers, Clinical Trial, Hispanic, Latin, Latinx

## Abstract

This study evaluated two types of barriers that the authors deemed important to resolve during the early stage of cancer clinical trial exploration by Latinx community members. One was the accessibility of information provided on cancer centers’ websites. The other was the telephone responders’ clinical trial knowledge and their conveyance of a warm welcome to Latinx callers inquiring about the centers’ clinical trials. Simulated clinical trial inquiry calls were made to 17 National Cancer Institute-designated centers in this study. The centers were located in cities where the Latinx community accounted for at least 25% of the population, thereby justifying center-wide efforts to encourage the Latinx community to explore clinical trial participation. A rubric was developed to determine and quantify a *Total Score* that was partially composed of the accessibility of clinical trial information displayed on each cancer center’s website. A research assistant gathered information by posing as a person calling the cancer center to inquire about clinical trials on behalf of a family member with limited English proficiency and evaluated their response using a “mystery shopper” method of data collection. The warmth and sense of welcome conveyed by the telephone responder was also quantified and included in the rubric’s *Total Score*. A perfect *Total Score* reflected the likely existence of an environment that would encourage Latinx community members to continue exploring clinical trials, i.e., removed or diminished possible barriers. Welcoming characteristics, such as those elements included in the scoring rubric, can be monitored regularly to assure that centers are consistently conveying an optimal sense of welcome to the Latinx community, while also providing accessible clinical trial information. Among the 17 cancer centers, no correlation was found between the size of the Latinx population served and each center’s *Total Score*.

## Introduction

President Joseph Biden’s Cancer Moonshot Initiative recognized that rapid advances in cancer discovery are driven by clinical trial research (https://www.cancer.gov/research/key-initiatives/moonshot-cancer-initiative). Participant diversity in clinical trials is critical because studies have shown that people of different ancestry and sociodemographic characteristics can have different health outcomes even when the same prevention and treatment plans are recommended. Thus, it is crucial for research studies to enroll participants of diverse characteristics when testing the safety and efficacy of promising medical discoveries to confirm the generalizability of the findings. Research conducted with samples that are not representative of the nation’s communities creates findings that cannot be generalized with confidence to all members of the population. In spite of this, research findings are still applied as if they had been proven effective for all population subgroups [1]. Thus, inadequate sample diversity becomes a clinical as well as an ethical problem, because it can contribute to suboptimal health care and health outcomes, which can lead to health disparities.

The Revitalization Act of 1993 sought to address this problem. This Act required that clinical trials funded by the National Institutes of Health must be conducted with participant samples that include representative numbers of women and members of minority groups [2, 3]. Despite this mandate, the scientific literature is replete with examples of non-representative study samples and evidence of barriers that members of minority communities encounter in relation to clinical trial participation. These studies report such barriers as mistrust of the research community, language barriers, time constraints, and lack of awareness of, and knowledge about, clinical trials [1, 4, 5]. Perhaps the best example of these barriers begins with a review of ClinicalTrials.gov. The website is difficult for a lay person to navigate, contains complex vocabulary, and is not available in Spanish or other languages besides English. The site is best explored in collaboration with a cancer care provider’s navigation assistance.

Despite these known barriers, research shows that members of the Latinx community in the USA, for example, are just as willing to participate in clinical trials as their non-Latinx White counterparts [6]. This study explored whether additional barriers present at the point of early inquiry could contribute to underrepresentation of the Latinx community in clinical trials.

## Methods

This qualitative study was reviewed by the authors’ Institutional Review Board, which determined that human subjects’ approval was not required since no personal data were being collected. The study was conducted in 2017 as a summer project for two undergraduates funded by a National Cancer Institute (NCI) research education grant focused on reducing cancer disparities. This student-led project focused on an evaluation of the accessibility of clinical trial information on cancer centers’ websites. It also evaluated the responses given to a simulated Latinx caller who was inquiring about clinical trial participation for a family member with limited English fluency, using the centers’ publicly accessible telephone numbers.

This study’s goals were to evaluate (1) the public accessibility of a cancer center’s information about clinical trial participation and (2) whether Latinx community members experienced a sense of welcome from the center when exploring clinical trials. To begin evaluating related entry point barriers to clinical trial exploration, the researchers opted to contact the NCI-designated cancer centers. The NCI expects its designated cancer centers to have representative samples in their clinical trials. Thus, NCI-designated centers were cancer research venues anticipated to model a high standard of excellence in the recruitment of diverse participants to their research studies.

To gather the data needed to evaluate a cancer center’s success at accomplishing both goals, the researchers selected a mystery shopper approach. Mystery shoppers are commonly used in commercial market research to evaluate customer satisfaction, and they are increasingly being used to evaluate client satisfaction in health care settings. Hamlyn and colleagues used mystery shoppers to identify and quantify barriers faced by patients seeking to make a first consultation appointment at NCI-designated cancer centers [7]. Another study by Marks and colleagues used secret shoppers to assess facility-level acceptance of Medicaid insurance among patients diagnosed with cancer [8].

In the summer of 2017, there were 51 NCI-designated comprehensive cancer centers. This study tested the hypothesis that NCI-designated cancer centers located in cities with a Latinx population of at least 25% of the general population (based on the 2010 Census data) would demonstrate a welcoming attitude and easy accessibility to information about their clinical trials for members of the Latinx community. Those centers were also anticipated to be likely to offer the linguistic accommodations needed to enable members of the Latinx community to make a more fully informed decision regarding the further exploration of clinical trial participation. For this study, 17 centers located in 13 cities across nine states qualified for inclusion.

The two undergraduate students learned to identify, qualify, and quantify cancer disparity issues, their consequences, and possible solutions. One student was a bilingual member of the Latinx community, and the other student was a member of the Pacific Islander community. Together with their faculty mentors, they identified specific, clearly defined evaluation criteria to assure that all centers were evaluated using the same criteria and in the same sequence.

Table 1 displays the key metrics used to qualify and then quantify the presence of a cancer center’s welcoming attitude and provision of clinical trial information relevant at this early point of clinical trial exploration.

**Table 1:** The scoring rubric

	Criteria	Selection	Points
Website evaluation	Website: general information	Yes	2
		No	0
	Website: main contact number	Yes	2
		No	0
	Website: clinical trials page	Yes	2
		No	0
	Website: clinical trials direct contact number	Yes	2
		No	0
Phone evaluation	Automated phone system with Spanish option	Yes	1
		No	0
	Number of calls to reach 1st responder	1	3
		2	2
		3	1
		N/A	0
	Awareness of participation in clinical trials for monolingual Spanish speaker	Yes	2
		No	0
		U/S	0
	Awareness of Spanish interpreter availabilities	Yes	2
		No	0
		U/S	0
	Sense of welcome	Yes	3
		No	0
		C/E	0

*N/A* never answered, *U/S* unsure, *C/E* cannot evaluate

### Website Evaluation

Upon arriving on the cancer center’s homepage, the evaluators independently assessed each website’s sense of welcome as it might be perceived by members of the Latinx community. Prior research showed that photographic images presented without racial or ethnic qualifiers were unreliable indicators of Latinx ethnicity [9]. Therefore, visual representations of the Latinx community on the website were not deemed adequate for accurate evaluation. Instead, the evaluators searched for the presence of a general cancer center phone number, whether the website offered information about clinical trials in English and Spanish, and if a phone number was offered for more clinical trial information.

### Telephone Evaluation

**Fig. 1:**
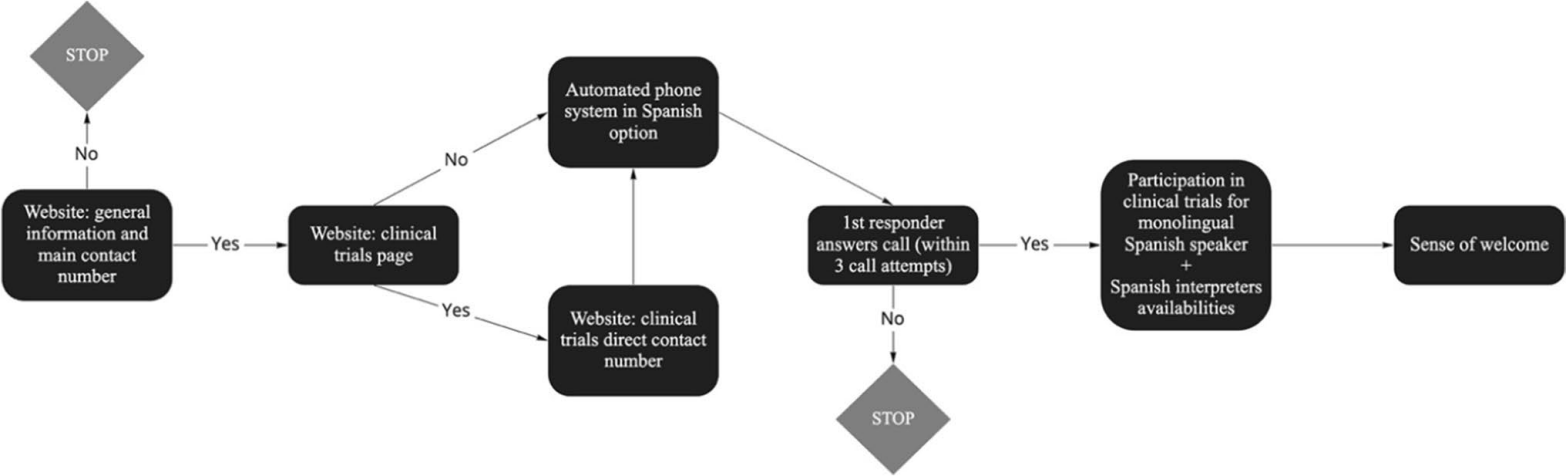
Flowchart of methodology

Calls were made directly to the cancer centers’ clinical trial line, whenever that number was provided on the centers’ websites. Otherwise, calls were made to the cancer centers’ main telephone number. All calls were placed during normal business hours in each center’s time zone. A successful call was defined as one that resulted in immediate, direct contact with a “responder” from the cancer center or contact with a person following a series of automated choices designed to lead the caller to an optimal first responder (see Fig. 1). If a call was not connected to a responder who could answer questions, no messages were left. A second call was placed on the subsequent business day. If the second call was not answered, a third and final call attempt was made. The number of calls placed to reach a first responder was recorded. The centers’ response to each call was also scored on whether the phone tree offered the option of accessing an automated system in Spanish.

Once contact was made with a responder, a semi-structured script guided each audio-recorded phone interaction. The simulation scenario was conducted by the study’s bilingual English/Spanish-speaking Latinx female. She called each center using the phone number provided on the cancer center’s website. She explained that she was calling on behalf of a relative who had heard about clinical trials but was concerned that English proficiency might be a requirement to participate. Acting as a family member, she inquired if Spanish-speaking staff would be available to help with translations if her relative participated in a clinical trial. The responders’ answers were recorded and independently scored by the evaluators.

The centers’ telephone accessibility for the Latinx community was further scored on the responder’s conveyance of a sense of welcome to the caller and her relative and appreciation of their interest in clinical trial participation. These welcoming attributes during the phone call included attitude, tone, word choice, patience addressing the caller’s questions, genuine effort to help the person gain access to the clinical trials office and information, and appreciation for the caller’s interest in seeking clinical trials information.

### Scoring Centers’ Overall Sense of Welcome to Clinical Trials Exploration by the Latinx Community

The same bilingual English/Spanish evaluator placed all the calls, and both evaluators listened to each live phone call. They independently used a rubric that was developed to review and score each of the categories evaluated during and immediately after each phone call (Table 1). The calls were recorded to facilitate reaching consensus with confidence on those rare occasions when scores differed.

Most criteria were scored with either zero (did not meet the criteria) or two (met the criteria). There were three scoring exceptions (Table 1) where the research team unanimously agreed that the characteristics being scored required a more refined scoring option to recognize the subtle variable degrees of responses.

## Results

### Website Evaluation

A possible total of eight points could be earned from the website evaluation. Of the 17 cancer centers, all the centers had a website that included general information and a main contact number. In addition, all the cancer centers had a clinical trials page. While 14 of the 17 centers had a direct contact number for clinical trials, none of the websites promoted a telephone number for Spanish speakers.

### Telephone Evaluation

In the absence of a dedicated phone number for Spanish speakers, a possible total of clinical eleven points could be earned from the telephone evaluation. Upon dialing the main number, only five of the 17 cancer centers had the option of an automated phone system in Spanish. After all the calls were made, it took three calls to reach the responder for nine centers and two calls to reach the responder for three centers. Only four centers required just one call to reach the responder. Phone calls to one center were not answered after three attempts.

Fifteen of the centers’ first responders provided useful information related to trial participation for monolingual Spanish speakers. Fourteen of the centers’ responders shared information about the availability of a Spanish interpreter. Of the 16 centers at which a responder was reached, 11 were awarded points for their responders’ clear conveyance of a sense of welcome to Latinx community members.

### Cumulative Scores

The data from the scoring of the website and the telephone evaluations were combined to create a *Total Score*. It was hypothesized that all cancer centers in cities with a Latinx community of at least 25% of the population would be motivated to create an environment that would encourage and facilitate Latinx community members’ exploration of clinical trials.

**Fig. 2:**
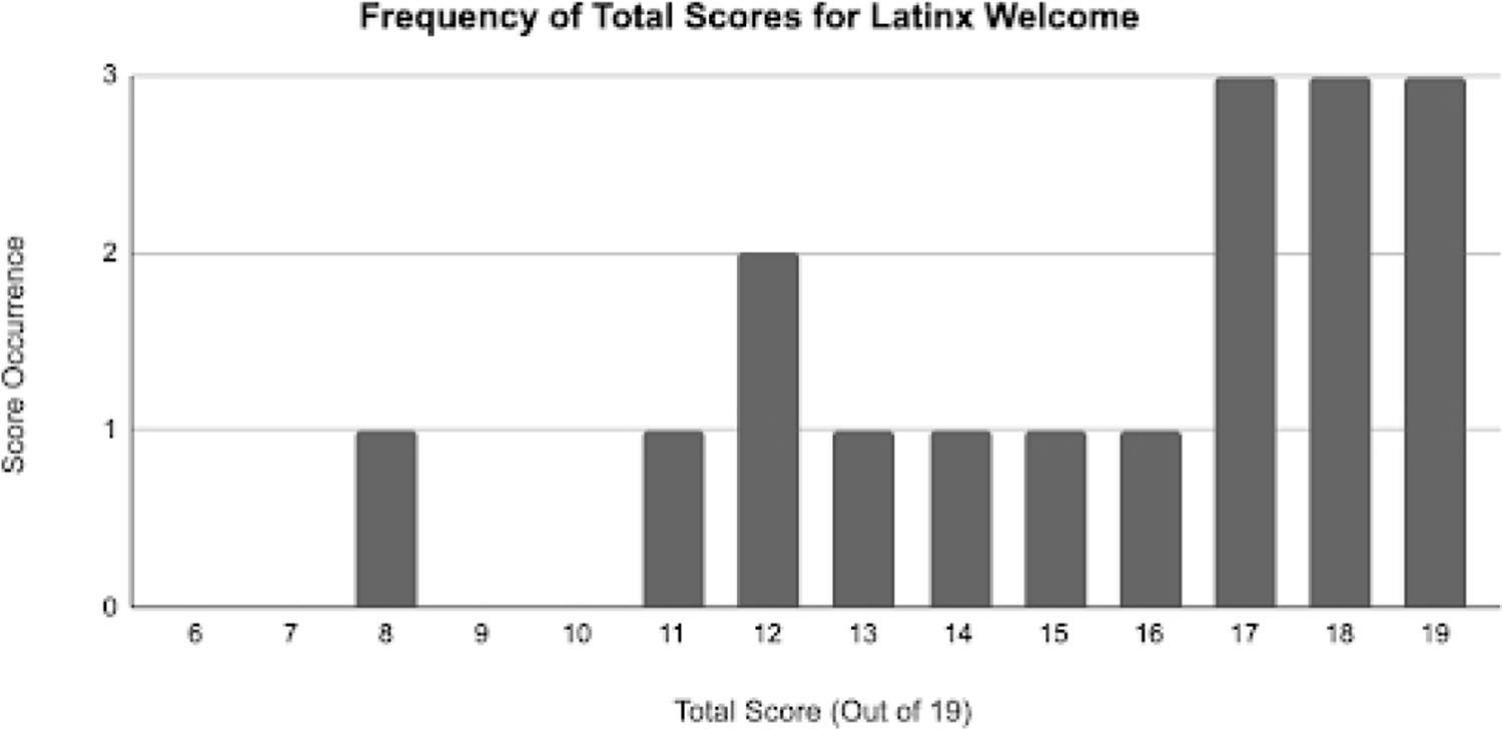
Frequency of total scores for Latinx welcome

Figure 2 shows the frequency of the *Total Scores* earned when each center’s points were summed across the website and telephone evaluations. While none of the centers promoted a dedicated line for Spanish speakers, three centers demonstrated all the other attributes considered valuable in helping the Latinx community explore clinical trial participation. These three centers’ achievement of a near-perfect Total Score suggested that the Latinx community members would likely feel welcomed and appreciated for their exploration of clinical trials at those centers and receive the information needed to encourage further exploration of clinical trials. No correlation existed between the size of the Latinx community served by the centers and the *Total Scores* of the cancer centers.

## Discussion

Clinical trial results that arise from a well-diversified sample of participants yield findings that can be generalized with greater confidence. Accruing well-diversified samples is challenging; dynamic efforts are needed to create such samples. Positive first impressions are critical when people are beginning their exploration of clinical trial participation, making it essential for cancer centers to address potential barriers early in the clinical trial exploration process. Cancer centers in cities with even a modest Latinx population have the opportunity to help increase the Latinx community’s clinical trial participation.

This research project focused exclusively on identifying the easily remediable barriers that Latinx community members might encounter during the early stages of their clinical trial exploration. The near-perfect scores earned by three of the centers demonstrated that the barriers selected were achievable performance goals.

These barriers are universal to all groups that are underrepresented in clinical trials. Cancer centers can adapt this study’s methods to evaluate their own center’s success in providing easy access to clinical trial information and a warm welcome to all underrepresented communities within their own center’s catchment area.

The authors ultimately decided on focusing on ten relatively simple criteria which they anticipated all centers could reasonably be expected to demonstrate and, through them, to reflect an institution’s sense of welcome toward the Latinx community. This list can be customized with additional criteria unique to a center. For example, cancer centers with larger Latinx communities could create an entire website with content comparable to their English language website. The translated pages could include a phone number that offers a return call from a Spanish speaker.

Customized websites might also include a link to generic Spanish language clinical trial education programs, such as those offered by the NCI or the American Cancer Society. Links could also be provided to community, regional, state, and national organizations that endorse the participation of the Latinx community in clinical trials. These links can help to create a sense of trust in a center that provides equivalent access to clinical trial information for the Latinx community.

An initial welcoming response sets the stage for the caller to ask more questions and to pursue clinical trial participation with more refined knowledge of how to proceed. If the first line of access to information is not warm and welcoming, then any welcoming efforts beyond the frontline may never be experienced by the person making the initial inquiry. Thus, the first line responders play a critical role in welcoming their Latinx community members and making it easy for them to continue exploring the feasibility of enrolling in a clinical trial.

This study demonstrated that first responders to telephone inquiries from Latinx community members could be better trained. First responders need to know: how to make callers feel valued and welcomed; more about clinical trials in general and where to direct callers for more information; how to process inquiries about their own centers’ clinical trials; the websites and phone numbers to give callers regarding their centers’ clinical trials; and, most importantly, information that monolingual Spanish-speaking people will need to know, such as details related to the accessibility of Spanish interpreters. For centers that cannot consistently staff their phone lines with a Spanish-speaking responder, telephone systems can triage monolingual callers to a message center with a welcoming statement in Spanish that promises a response from a Spanish speaker to those who will leave a phone number or email.

Evaluations of such welcoming criteria can be done periodically by cancer centers to assess whether desired improvements have been attained and sustained. It is preferable to have these simulated inquiries and other evaluations conducted by teams of well-trained Latinx staff and community members to assure that the most accurate assessments are being made on behalf of the center and the community. Community-campus partnerships can help cancer centers’ responders to understand their local Latinx community’s and other communities’ perspectives of what constitutes a “warm and welcoming” responder.

### Limitations of the Study

These evaluations were conducted by a single two-person team. It is possible that a different two-person team with different socio-cultural attributes and experiences might have evaluated the human responses differently. Also, this was a one-time only evaluation. Results may vary with different responders or repeated, periodic evaluations of the same responder. In hindsight, this study could have evaluated whether the website contained a discussion about the importance of sample diversity and whether that was provided in English and Spanish. Finally, this data collection was completed in the summer of 2017, so the findings may not represent a more recent evaluation.

### Recommendations for Future Research

Evidence-based training programs are needed to help centers’ first responders engage more effectively with callers inquiring about clinical trial participation. Such training programs would promote first responders’ consistency across cancer centers and do so with optimal efficiency. Training needs to include the most basic training of the first responders’ job, plus more advanced training on clinical trials. Practice evaluations with simulated callers in English and Spanish will enable centers to meet their Latinx community’s clinical trial information needs better, while also potentially helping to expand the diversity of the volunteers participating in their centers’ clinical trials.

## Conclusions

This study explored multiple points that could discourage Latinx community members from continuing their preliminary exploration of clinical trials participation. Once such problems are identified, a center will likely be able to resolve those barriers. The same methods used for the evaluations conducted in this study for the Latinx community can be readily adapted for centers that serve other communities that are underrepresented in clinical trials.
